# Preferences for Chronic Pain Treatment Among Indigenous Peoples Living in the Pacific Northwest

**DOI:** 10.3390/ijerph23040502

**Published:** 2026-04-14

**Authors:** Andrea K. Newman, Mark P. Jensen, Kara Link, Kathy Littlebull, Molly Fuentes, Chantelle E. Roberts, Robin John, Ryan G. Pett

**Affiliations:** 1 Department of Anesthesiology, University of California, San Diego, CA 92093, USA; 2 Department of Rehabilitation Medicine, University of Washington, Seattle, WA 98104, USA; 3 Department of Psychology, University of New Mexico, Albuquerque, NM 87131-0001, USA; 4 Portland Area Indian Health Services, Yakama Service Unit, Toppenish, WA 98948, USA

**Keywords:** chronic pain, indigenous health, community-based

## Abstract

**Highlights:**

**Public health relevance—How does this work relate to a public health issue?**

- Chronic pain prevalence is significantly higher in Indigenous populations than in other groups.
- There is a critical lack of culturally grounded and culturally appropriate chronic pain treatments tailored to the needs and values of Native communities.

**Public health significance—Why is this work of significance to public health?**

- This study assesses interest in psychological treatments for chronic pain among Indigenous community members receiving care for chronic pain from the Portland Area Indian Health Services—Yakama Service Unit.
- The findings inform the development of future culturally adapted psychological pain interventions aimed at addressing severe and persistent pain disparities.

**Public health implications—What are the key implications or messages for practitioners, policy makers and/or researchers in public health?**

- Indigenous peoples receiving care from the Portland Area Indian Health Services—Yakama Service Unit are interested in psychological approaches to pain management.
- Focus group discussions identified pain intensity and pain interference—particularly social interference—as the most important outcome domains to prioritize in developing a culturally adapted psychological pain treatment.

**Abstract:**

There is a significant need for culturally appropriate psychological treatments for chronic pain among American Indian/Alaska Native (AI/AN) peoples. This study used Indigenous community-based participatory research methods with the Portland Area Indian Health Services—Yakama Service Unit (YSU) to gather information needed for developing culturally adapted psychological treatments for AI/AN individuals with chronic pain. This study included remote semi-structured focus groups with 16 AI/AN individuals with chronic pain to identify pain treatment preferences (Aim 1) and priorities for pain treatment outcome domains (Aim 2). Thematic analyses were conducted with Atlas.ti (version 23.2.1). Results indicated a high interest in psychological interventions and concern that referral to psychological treatment meant that pain is “not real.” Pain intensity and pain interference were identified as the most important outcome domains. To measure pain intensity, the 0 to 10 Numerical Rating Scale was most preferred. The findings support the potential utility of culturally adapted psychological treatments for chronic pain for AI/AN individuals and provided information regarding the adaptations that would be most useful.

## 1. Introduction

American Indian/Alaska Natives (AI/ANs) have among the highest prevalence rates of chronic pain [1]. Given historical traumas—including systematic genocide, colonization, forced relocation, and boarding school placement—and continued experiences of trauma and adverse events, Native populations experience a higher-than-average risk of developing chronic pain [1,2,3]. Further, AI/AN individuals with chronic pain experience an almost three times greater likelihood of having high impact pain compared to chronic pain without limitations [4]. During the crisis of opioid-related deaths in the USA, Native populations experienced the largest increases in opioid-related mortalities than any other racialized/ethnic groups [5]. In addition, AI/AN adults may experience higher levels of pain-related anxiety and catastrophizing, likely contributing to the increased risk for chronic pain and related suffering [6].

Given the high levels of disparities, research is needed to develop culturally appropriate pain interventions for AI/AN communities. Common treatment barriers for Indigenous peoples in the USA include limited health service access, use, and coverage [7,8], lack of culturally congruent care [3], invalidation of pain complaints from providers [9], provider stigma of opioid addiction as a barrier to treating pain [10], and patient-experienced barriers in financial constraints, transportation, and provider availability [3].

Moreover, the treatment options available are often limited to biomedical treatments which conceptualize chronic pain in primarily biological terms, typically focusing on medications and procedures. Unfortunately, biomedical treatments alone are often ineffective, are associated with significant risk for adverse events, and do not represent the current standard of care for chronic pain [11,12,13]. Research has also found that AI/AN individuals with chronic pain tend to report concerns of risks of medicines [10] and a non-biomedical conceptualization of pain in which pain is conceptualized in the context of physical, emotional, social, and spiritual aspects [14]. Historical and ongoing experiences of discrimination and trauma within health care systems have shaped medical mistrust in many AI/AN communities, underscoring the need for culturally appropriate pain interventions.

Psychological treatments, which target the cognitive, emotional, behavioral, and social factors that influence the experience of pain, are well-established as effective for chronic pain management, are considered an integral part of multidisciplinary chronic pain management, and are associated with few significant adverse events [15]. However, there is a lack of evidence-based culturally adapted psychological pain interventions for AI/AN populations. Culturally congruent care refers to care that is aligned with community-specific cultural values, beliefs, and ways of understanding health and healing [16]. Without cultural adaptation and community guidance, pain treatments may be less effective, less sustainable, and unintentionally reinforce stigma or invalidate patient experiences. The goal of the current study was to take the first steps needed to develop a psychological chronic pain intervention tailored to the specific needs of Indigenous adults in the Pacific Northwest receiving care for chronic pain from the Portland Indian Health Services—Yakama Service Unit (YSU). To address this goal, we conducted qualitative focus groups with Native individuals with chronic pain served by the YSU, with the specific aims to: (1) identify the pain management techniques preferred by community members, and (2) identify the outcome domains and measures of the outcome domains that were viewed as most important and culturally appropriate by community members. Addressing the first aim is important for developing a treatment that will be of interest and well-accepted. Addressing the second aim is important for the identification of treatment targets, and the initial evaluation of the cultural appropriateness of potential outcome domains to evaluate treatment effects. Understanding which outcomes are most important to community members allows for the development of treatments that intentionally target those outcomes and for the evaluation of efficacy using culturally acceptable measures.

## 2. Materials and Methods

### 2.1. Setting

The Portland Area Indian Health Service—Yakama Service Unit (YSU) is an accredited health care facility located on the Yakama Nation Reservation run by the Indian Health Service that provides health care to all eligible American Indians and Alaska Natives. Yakama Nation has also welcomed Native individuals from various Tribal communities, creating a diversity of Indigenous individuals and families. There is a significant poverty rate of 19% [17], likely suggesting various barriers to pain treatment access. Challenges in accessing specialized pain management are significant, exacerbated by local shortages in health care professionals and facilities. Unlike Tribes composed of a single ancestral group, the Yakama Nation is a confederation of 14 Tribes and bands that were consolidated through the Treaty of 1855, resulting in a large, culturally diverse Tribal population. The Yakama Reservation is not only geographically expansive, but is welcome to AI/AN individuals from other Tribal Nations. This contributes to a rich cultural landscape with a variety of traditions and practices.

### 2.2. Involvement of Community Stakeholders in Study Design

Providers of the YSU partnered with University of Washington researchers to address community health needs for non-pharmacological chronic pain interventions. Using an Indigenous community-based participatory research approach, we designed a qualitative study to gather initial information needed to develop culturally adapted psychological treatments for chronic pain at the YSU. To develop the study design, a project advisory board was created which included YSU physicians, nurses, pharmacists, administrative staff, behavioral health providers, and Tribal Council members. Multiple meetings, emails, and phone calls were conducted to identify the study aims, the roles and responsibilities of stakeholders, and the design of the research project, as well as to address ethical issues and discuss and disseminate the study findings.

### 2.3. Participants

Potential study participants were identified using a combination of random and purposive sampling through a review of medical records at the YSU by clinical providers. First, a random list of patients with documented chronic pain diagnoses was generated. Providers then reviewed this list and purposively selected individuals based on their clinical knowledge of patients’ likelihood of interest and ability to participate meaningfully in a group-based qualitative study (e.g., communication style, reliability for follow-up, and current health status), with the goal of maximizing engagement and the richness of data while minimizing participant burden. All patients at the YSU are American Indian or Alaska Native and are affiliated with at least one tribal community. The review of medical records identified individuals with at least one chronic pain diagnosis. Recruitment aimed to have an equal ratio of men and women. When the initial random list of potentially eligible patients was generated, providers were asked to be attentive to participant sex during the purposive selection process. As recruitment progressed, providers prioritized inviting individuals from the underrepresented sex to subsequent focus groups when feasible, while maintaining the primary inclusion criteria related to chronic pain diagnosis, likelihood of participation, and ability to engage in group discussion. This approach was intended to support a balanced representation of perspectives while remaining responsive to practical considerations such as availability, interest, and participant burden. Providers initially contacted potential study participants by phone. If interested, study participants were then contacted by phone for screening by the PI. Inclusion criteria included self-identification as an American Indian or Alaska Native, at least 18 years of age, and with a diagnosis of chronic pain as per their medical chart. There were no exclusion criteria.

### 2.4. Measures

A brief semi-structured interview was conducted to ask about the participant’s pain diagnosis, pain location, and pain duration. We chose an interview approach to facilitate conversation and allow participants to share their narratives with chronic pain, consistent with suggestions from prior research and as recommended by the advisory board for this project [18].

We assessed pain intensity and pain interference for the purpose of describing the study sample. Pain intensity was assessed using the 3-item Patient Reported Outcomes Measurement Information System (PROMIS) Short Form 3a (Version 2.0)—Pain Intensity scale [19]. Study participants reported average pain intensity and worst pain intensity over the past 7 days and current pain intensity on a 5-point verbal rating scale, including: No pain (1), Mild pain (2), Moderate pain (3), Severe pain (4), and Very severe pain (5).

Pain interference was assessed using the 4-item PROMIS Short Form 4a (Version 1.1) —Pain Interference scale [20]. With this measure, respondents rate the extent to which pain interfered with different activities over the past 7 days using a 5-point Likert scale, including: Not at all (1), A little bit (2), Somewhat (3), Quite a bit (4), Very much (5).

The PROMIS Pain Intensity and Pain Interference short form scales have been recommended for use in chronic pain populations by the NIH Chronic Low Back Pain Research Task Force [21] and have been shown to have acceptable to excellent internal consistency [22]. The internal consistency (Cronbach’s alpha) of the PROMIS Pain Intensity and Pain Interference scales in the current sample were 0.84 and 0.87 respectively, indicating good reliability.

### 2.5. Procedures

This study was approved by the University of Washington Institutional Review Board, the Portland Area Indian Health Services Institutional Review Boards, and the Yakama Nation Tribal Council. Due to concerns for safety related to COVID-19, and in order to maximize participation, all focus groups were conducted via a HIPPA-compliant Zoom platform. Participants without access to the internet or a device with video conferencing capacity were provided hot spot devices and tablets for the duration of the study. The qualitative focus groups were co-led by the PI and a co-facilitator who identified as American Indian and was from the community. The PI was a female post-doctoral fellow with a PhD in clinical health psychology and the co-facilitator was a female registered nurse at the clinic. The PI received training in qualitative research through graduate learnings. Prior to study commencement, the PI did not have contact with any study participants; however, the co-facilitator may have provided clinic health services to some study participants. As a non-Indigenous investigator, the PI recognizes that her professional background and social positionality may have influenced the study design, group discussion, and interpretation of the data. To mitigate the potential bias and strengthen cultural accuracy and relevance, the following strategies were implemented: (1) co-facilitation with an Indigenous community member, (2) consultation with the project’s advisory board, and (3) member-checking procedures throughout focus group sessions and with one study participant during post-data analysis.

Open-ended semi-structured questions were used for all focus group interviews. The questions were developed in collaboration with the co-facilitator and the community advisory board. A focus group moderator guide was used. Each focus group lasted about 1.5 h and participants were compensated at a rate of $30 per hour. Focus groups took place from November to December 2022. The co-facilitators took notes during sessions, reflected back themes and summaries to the group, and checked for accuracy of these reflections during the interview with group members. Sessions were audio-recorded and transcribed.

Six focus groups were conducted with two to eight individuals per group. Participants were assigned to focus groups primarily according to scheduling availability, with secondary consideration given to balancing participant sex across groups. In accordance with suggestions for culturally competent interviewing techniques, focus groups began with the invitation to share life narratives before asking questions [18]. To address Aim 1, three focus groups (*N* = 16 AI/AN adults with chronic pain) were conducted that focused on preferences for pain management techniques. The questions elicited discussions regarding prior techniques used for pain management, what is missing from current pain care, interest in additional pain management techniques, and perceptions of the term “psychological treatments for chronic pain” (see supplemental attachment for full list of questions). The PI provided descriptions of psychological techniques for pain management (i.e., mindfulness, behavioral, cognitive, and hypnotic techniques) and asked study participants for their thoughts about each technique, including interest in learning more about and potentially using these techniques. The group co-facilitators also explored preferences for how pain management techniques might be taught (e.g., individual vs. group, in-person vs. telehealth, time of day, location, and number of sessions).

To address Aim 2, three focus groups were conducted with the same study participants. The topics discussed in these focus groups were priorities for pain management outcomes and the interest in and/or concerns about specific measures that could be used to assess those outcomes. First, group facilitators asked study participants to discuss the treatment outcomes that were more important to them. Study participants were not offered a list of outcome domains, but rather were asked to identify outcomes of greatest importance independently. Next, the group co-facilitators described specific measures that could be used to assess the most preferred outcomes and elicited feedback about those measures regarding their acceptability, clarity, and cultural congruence.

The transcripts from the focus groups were analyzed using Atlas. Ti (version 23.2.1) [23]. First- and second-cycle coding procedures were used as part of an inductive analytic process, moving from initial descriptive coding to pattern-based thematic development to create a working list of themes, which were then organized into thematic codes [24]. The themes that emerged were reviewed by the PI and an additional UW researcher trained in qualitative analysis and included listening to the recorded focus groups and reading the transcripts. Intercoder agreement was consensus-based: coders compared coding, discussed discrepancies, refined the codebook, and reached agreement through discussion rather than statistics. When disagreements arose, coders revisited the relevant transcript segments, examined them in relation to the study aims and codebook, and refined code definitions as needed. The accuracy of results was further validated through a review with the co-facilitator and member checking with one study participant from the focus groups. Although member checking was planned with three participants, two individuals were unable to attend the scheduled member-checking session.

## 3. Results

### 3.1. Overview

We initially contacted 23 Native adults with chronic pain identified from medical records. There were four individuals who were either not interested in the study, too busy, or whose phone number was no longer in service. We screened and consented 17 Native adults with chronic pain. While all 17 agreed to participate, one individual withdrew prior to the focus groups being conducted due to a busy schedule. Data collection and analysis occurred iteratively, with an ongoing review of emerging themes. Recruitment ceased once thematic saturation was reached, as later focus groups did not yield new themes or meaningfully expand existing categories. The final sample size was considered adequate for the study aims, as qualitative research tends to focus on in-depth exploration of experiences, prioritizing informational richness and thematic saturation over large sample sizes. The number of participants and focus groups was sufficient to capture shared patterns as well as meaningful variation in perspectives among Indigenous adults with chronic pain receiving care at the YSU.

Please see Table 1 for a description of study participants. Participants reported moderate to severe levels of pain intensity [25] and pain interference [24]. All participants reported more than one chronic pain condition, with chronic back pain as one of their pain conditions. Nine participants (56%) reported back pain to be their primary or co-primary pain problem. Ten participants (63%) reported a diagnosis of arthritis.

Thirteen of the 16 study participants (81%) attended focus group sessions related to Aim 1 and 10 study participants (63%) attended focus group sessions related to Aim 2; 10 participants (63%) attended both an Aim 1 and an Aim 2 focus group.

### 3.2. Aim 1—Chronic Pain Treatment Approaches

**Figure 1:**
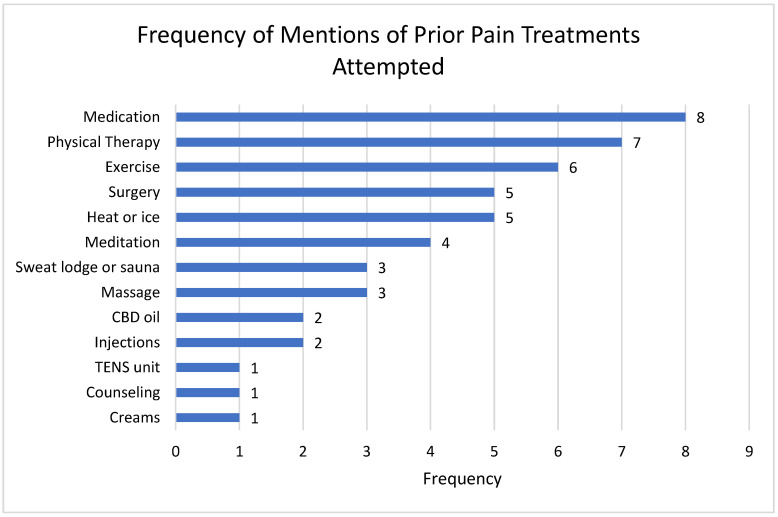
Number of times participants mentioned trying prior treatments for pain management.

Qualitative focus groups first explored prior treatments used for chronic pain. The most mentioned previously attempted methods for managing chronic pain were physical therapy, analgesic medications, and exercise (see Figure 1). In terms of psychosocial treatments, four individuals had tried meditation, one tried counseling, and one had used sweat lodges for pain management. In terms of interest in trying treatments in the future, participants mostly mentioned body-oriented treatments, such as exercise, sauna, massage, and invasive biomedical procedures (i.e., nerve ablation or cortisol injections).

After providing descriptions of mindfulness, behavioral activation, cognitive, and hypnotic interventions, participants expressed interest in and an openness to trying psychological pain treatments (see Table 2 for representative quotes). One participant said, “Having someone who really knows about the psychological side of dealing with chronic pain would probably be a real benefit.” Another mentioned the need for psychological treatments due to the risk of depression and suicide with chronic pain. Across mindfulness, behavioral, and cognitive treatments, there was an equal spread of interest, neutrality, and disinterest/skepticism. There was a higher level of uncertainty with therapeutic hypnosis.

In terms of a treatment structure, the participants expressed variable preferences related to timing, number of sessions, in-person vs. remote, and group vs. individual treatment; no single type of treatment approach in any of these categories emerged as a clearly preferred approach. Obstacles related to timing included the winter season in which snow in Western Washington could prevent clinic accessibility. A smaller number of sessions (i.e., 3–4 sessions) was generally preferred due to the numerous obstacles in treatment access. About half of the study participants preferred in-person and half preferred remote treatment. There was also an even split in preference for group versus individual treatment. In addition, while there was no consensus regarding preference for treatment providers (i.e., the treatment providers could be peers, mental health providers, or medical staff), it was clear that participants wanted treatment providers who are knowledgeable about chronic pain and who would be non-judgmental and validating. Another common theme was a desire to have family involved in treatment, which could make treatment more culturally congruent and potentially effective.

### 3.3. Aim 1—Sub-Theme: Stigmatization and Invalidation

Concerns about stigmatization and invalidation related to experiences with chronic pain emerged as a common sub-theme. Almost all the participants noted or confirmed similar experiences of being treated unfairly, labeled negatively, misunderstood, and/or not being believed to truly have chronic pain (mentioned 15 times across the three focus groups; see Table 3 for representative quotes). Many participants mentioned that they had been labeled as a “drug addict” or that they were perceived by providers to be drug seeking. Many also mentioned that they had not felt heard, that their health care providers thought that their pain was “not real”, or that the pain was “made up” or caused by anxiety or depression and therefore “all in the head.” Sometimes participants were referred to mental health services instead of (also) being evaluated and treated for pain by a medical provider. As a result of being referred to mental health treatment when seeking care for pain, participants felt unheard and not believed by providers. The stigmatization and invalidation occurred across multiple care settings (i.e., primary care, emergency care, urgent care) and was not specific to any one clinical setting.

The association between pain invalidation and mental health issues further led to a perceived stigmatization of psychologically based treatments for chronic pain. When participants were asked about their reactions to the term “psychological chronic pain treatments,” many commented on feeling perceived as being “crazy” or as “faking their pain.” The patterns of stigmatization and invalidation highlight important and necessary treatment development adaptations that are discussed in the conclusion section of this paper.

### 3.4. Aim 2—Outcome Domain Preferences

**Figure 2:**
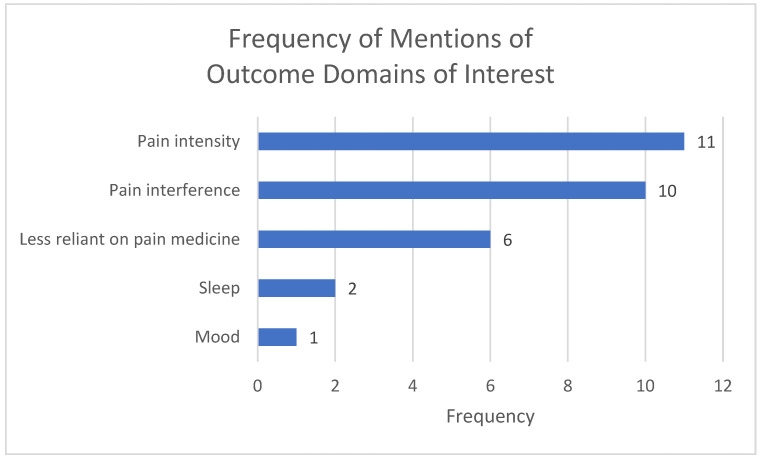
Number of times participants mentioned specific outcome domains of interest.

Most participants endorsed pain intensity and pain interference as outcome domains of primary interest (see Figure 2). The third most common outcome domain was feeling less reliant on analgesic medications. It is important to note that participants specifically mentioned not wanting “reduction in pain medicine” to be an outcome, but instead wanted the treatment to provide them with tools to manage pain which would then allow them to be less dependent on analgesic medications.

In order to assess preferences for measures of pain intensity, we showed the study participants examples of the four pain intensity measures used most often in pain research [26,27] the Visual Analog Scale (VAS) [28], the Faces Pain Scale—Revised (FPS-R) [29], the Numerical Rating Scale (NRS) [30,31,32], and the Verbal Rating Scale (VRS) [27,32]. All measures were screen-shared through Zoom on a PowerPoint slideshow. See Table 4 for description of all measures explored.

The majority of the participants preferred the 0 to 10 NRS over the other pain intensity measures. Study participants commented that they felt most comfortable with this scale because of their familiarity with it; their health care providers use this measure in their medical appointments. That said, the study participants also noted that they liked the VRS as well, commenting that they liked the descriptions and felt they were “… accurate to what [they] go through daily.” Most participants disliked the VAS and the FSP-R.

For assessing pain interference, we described and showed the participants two of the most common measures of pain interference: the 10-item Brief Pain Inventory (BPI) Pain Interference scale [33] and the PROMIS Pain Interference—Short Form 8a [34]. The results were mixed with respect to preferences for measuring pain interference. While some participants preferred the BPI and others preferred the PROMIS Short Form 8a, there was a slight preference from the PROMIS Short Form 8a due to the greater number of items that assessed interference with social activities due to pain.

Although measures of mood were not noted often as an outcome of interest, measures of negative affect are commonly used as secondary outcomes in pain clinical trials. This is due in part because psychological function domains (including anxiety and pain catastrophizing) are thought to mediate treatment outcomes and are often targets of psychological pain treatment [35,36]. Therefore, for additional exploratory purposes, we described and showed two measures of pain catastrophizing to the study participants—the Pain Catastrophizing Scale (PCS) [37] and the Concerns About Pain (CAP) scale (previously known as the Pain Appraisal Scale) [38,39].

Although some participants commented on how both scales were relevant and relatable, there were many participants who felt the questionnaires’ items made them feel vulnerable and were potentially threatening. For example, one participant stated, “I’m afraid… am I crazy if I answer it ‘Always,’ you know, or if I answer one of these questions as ‘Always,’ will they send me to the psych ward?” There were a few participants who said they would not feel comfortable responding to the questions honestly for fear of being diagnosed with a mental health condition, having a mental health diagnosis stay in their medical chart forever, and/or being referred to emergency mental health services.

**Table 4:** Description of each pain intensity and pain interference measure shown to study participants.

Measure	Description
Visual Analog Scale (VAS)	Measure of pain intensity. The VAS consists of one line in which respondents mark the point that best represents pain intensity from one end (“No pain”) to the other end (“Worst pain possible”) [28]. The distance from the “No pain” end to the park on the line (usually measured in mm) is the respondent’s pain intensity score.
Faces Pain Scale—Revised (FPS-R)	Measure of pain intensity. The FPS-R present drawings of 7 faces meant to represent different levels of pain intensity. Respondents are asked to select the face that best represents their pain intensity [29]. Each face is associated with a number (i.e., 0, 2, 4, 6, 8, or 10), and the respondent’s FPS-R score is the number associated with the drawing that was selected.
Numerical Rating Scale (NRS)	Measure of pain intensity. The 0-10 NRS is an 11-point scale from 0 to 10 where 0 indicates “No pain” and 10 indicates a very high level of pain (e.g., “Extreme pain,” “Pain as bad as you can imagine,” etc.) [30,31,32]. The respondent’s score is the number chosen.
Verbal Rating Scale (VRS)	Measure of pain intensity. The VRS consists of a list of adjectives reflecting increasing pain levels [27]. Respondents are asked to select the descriptor that best describes their pain intensity. Here we presented a 5-point VRS (i.e., “No pain” = 1, “Mild” = 2, “Moderate” = 3, “Severe” = 4, “Very severe” = 5) from PROMIS Scale v2.0—Pain Intensity 3a [27].
Brief Pain Inventory (BPI)—Pain Interference	Measure of pain interference. The BPI—Pain Interference consists of seven items assessing the extent to which pain interferes with seven activities on a 0 (“No interference”) to 10 (“Complete interference”) Numerical Rating Scale [33].
PROMIS Pain Interference	Measure of pain interference. Given the limitations in time, we only presented the eight items of the PROMIS Pain Interference Short Form 8a (rather than the full scale of 40 items) [34]. Respondents indicate the extent to which pain has interfered with the activity described by the item on a 5-point Likert scale (1 = “Not at all,” 2 = “A little bit,” 3 = “Somewhat,” 4 = “Quite a bit,” 5 = “Very much”).
Pain Catastrophizing Scale (PCS)	Measure of pain catastrophizing. The PCS includes 13 items reflecting different catastrophizing thoughts about pain. Respondents to this measure are asked to indicate the extent and frequency with which they have each thought when they experience pain on a 5-point Likert scale ranging from 0 (“Not at all”) to 4 (“All the time”) [37].
Concerns About Pain (CAP)	Measure of pain catastrophizing. The CAP consists of an item bank of 24 negative thoughts about pain developed using similar procedures used to develop the PROMIS item banks. As can be applied with the PROMIS item banks, investigators can select any number of items from the CAP item bank to create static scales. Here we showed the participants the six-item short form of the CAP. Respondents to the CAP items indicate the frequency with which they had each “concerning” thought about pain in the past 7 days on a 5-point Likert scale (1 = “Never,” 2 = “Rarely,” 3 = “Sometimes,” 4 = “Often,” 5 = “Always”) [39].

## 4. Discussion

### 4.1. Aim 1

There was an overall interest in psychological treatments for chronic pain. In terms of types of psychological treatments, there was wide variability in interest in each type, with a theme of more skepticism towards therapeutic hypnosis. Within clinical settings, there is generally high variability of interest towards different therapeutic approaches for chronic pain. Just as some people naturally navigate towards mindfulness, others may be more interested in cognitive treatment. It is likely that a mix of therapeutic approaches would offer the greatest potential for tailoring to individuals’ preferences and needs, and therefore for overall effectiveness. Treatments that involve family members and that involve an emphasis on social connectivity are likely going to be best received.

Prior to learning more about the available psychological treatments for chronic pain (as had been described by the PI), many study participants reported mistrust and skepticism. Given the stigmatization of and the low accessibility to psychological treatments for chronic pain across the United States, especially within underserved communities, mistrust and skepticism are common [40,41]. Within AI/AN populations, there has been a long history of racism, oppression, and colonization within the U.S. health care system that contributes to fears of stigmatization, including concerns that referral to psychological treatments for pain may signal disbelief in the legitimacy of patients’ pain experiences. Often, psychological pain treatments were perceived to be appropriate only for addressing mental health issues, and to be therefore only appropriate for people who are depressed, anxious, or who have “fake” pain. This is a common barrier to psychological treatments for chronic pain across all clinical settings and is often decreased through education on the biopsychosocial model of pain. Future intervention would likely benefit from provider training on how to most effectively communicate a biopsychosocial model of pain and ways to empathetically discuss psychological treatment as a key piece of effective multidisciplinary care for all individuals with chronic pain.

The significant narratives on pain invalidation are unfortunately common for individuals with chronic pain. However, the results implied levels of invalidation in the study sample that were higher than the average individual with chronic pain. Invalidation refers to a constellation of experiences of perceived disbelief, nonacceptance, suspicion of falsified or exaggerated symptoms, stigmatization, misunderstanding, or rejection by others [42,43]. This finding is consistent with the literature suggesting that experiences of pain invalidation may occur to a greater degree among minoritized communities [44,45]. Findings suggest the importance of addressing the patterns of invalidation within these clinical settings, such as through provider trainings and through treatments focused on healing from these negative social interactions.

Prior treatments for chronic pain reported by the study participants were mostly biomedical/body focused (e.g., medications, surgery, exercise, physical therapy, massage). It was not clear whether this finding was due to access issues (i.e., biomedical approaches are often the only ones available), interest (i.e., biomedical treatments being potentially of greater interest to the participants), or both. The expressions of interest in psychological treatments by the participants suggest the possibility that the former explanation is the more likely one.

Interestingly, the only mention of a traditional healing practice was the use of sweat lodges. The limited discussion around traditional healing practices contrasts with results to a prior study that showed high rates of traditional healing practices for chronic pain in one AI/AN community [46]. Because this study did not specifically probe for traditional healing practices, it may be that the study participants did not report practices such as using herbs and/or spiritual healing. The results may also reflect the loss of cultural practices due to colonization and emphasize the potential importance of integrating traditional practices into treatment in this population.

### 4.2. Aim 2

The findings from the focus groups conducted to address Aim 2 provided preliminary support for the use of common pain intensity and pain interference as primary outcome measures for chronic pain in clinical and research settings for individuals from the YSU community. Although study participants preferred the 0 to 10 Numerical Rating Scale for assessing pain intensity, there was also a positive reaction to the 1–5 PROMIS VRS of Pain Intensity. Similarly, both pain interference measures were acceptable to the individuals in the focus groups, although a major finding was a strong interest in assessing how pain interfered with interactions with family and other loved ones. In many AI/AN communities, family relationships are closely tied to cultural values and healing practices, with health and pain often understood and addressed within relational, family, and community contexts rather than solely at the individual level. Interventions that acknowledge and potentially involve family members may support healing by reinforcing social connection, reducing pain-related isolation, and strengthening community ties [47].

The findings suggested discomfort with assessments related to psychological function, such as concerns about pain or pain catastrophizing. Researchers should consider the possible negative effects of including measures of these domains. More information is needed to identify strategies for minimizing these negative effects; such strategies might include, for example, clarifying that responses will not go into their medical record and will not lead to a psychiatric diagnosis.

### 4.3. Limitations

The current study has a number of limitations that should be considered when interpreting the results. First, although the sample sizes for the two studies were appropriate for a qualitative study, the participants represented samples of convenience and may not have been representative of the population of individuals with chronic pain or all the health care providers in the YSU. Similarly, because participants came from different Tribes, there were not enough participants to be able to explore the effects of specific cultures and traditions originating from different Tribes on the findings. Also, the focus group questions did not specifically probe for the use of traditional healing practices for chronic pain. Early advisory board discussions suggested that the current study design was not well suited for exploring traditional healing practices. Instead, board members recommended that future research engage Tribal elders and community leaders, for whom this approach may be more appropriate, particularly given that some traditional knowledge and practices may be less accessible to broader community members due to the impacts of colonization. Due to time constraints, we also did not explore perceptions of measures related to depression, a common measure in clinical intervention research. The current findings suggest the possibility that questions related to depression, and in particular, questions regarding possible suicidal ideation, may be uncomfortable for the individuals with chronic pain treated by the YSU. Future research should continue to explore the appropriateness of measures of psychological function in AI/AN individuals in chronic pain research, as well as the best strategies for addressing any negative effects of the inclusions of measures of these domains.

### 4.4. Future Directions

Despite the study’s limitations the current study provides important new information that could inform the development and evaluation of culturally adapted psychological treatments for AI/AN individuals receiving care for chronic pain at the YSU. Future research would benefit from specifically examining sustainable, feasible, and acceptable pathways for implementing psychological services into existing care structures within Native communities, such as Indian Health Service (IHS) settings. At a policy level, these findings highlight the need for dedicated and sustained IHS funding to support behavioral health infrastructure for chronic pain care. It is important to note that without cultural adaptation and community guidance, psychological pain treatments may unintentionally reinforce stigma or invalidate patient experiences. These findings underscore the importance of workforce training policies that equip both medical and behavioral health providers with skills in chronic pain care, stigma reduction, culturally responsive communication, and the biopsychosocial model of pain. Consistent with qualitative research principles and this study’s aims, our conclusions do not generalize across other AI/AN populations. Future research should be conducted across various Tribal communities. Given the wide variety of cultures, traditions, and values across Tribal communities, it is likely that psychological treatments for chronic pain would need to be adapted in different ways to meet the needs of each Tribal community. Our long-term goal is to develop a template that could be useful for treatment adaptations in different communities across the country.

## 5. Conclusions

The study findings indicate that psychological treatments are of interest to Native adults with chronic pain receiving care from the YSU. Cognitive, behavioral, and mindfulness-based treatments were equally of interest; although there was some interest in therapeutic hypnosis, there was a higher level of skepticism and mistrust for this approach. Focus groups identified pain intensity and pain interference as outcome domains of greatest importance. In measuring pain intensity, the 0 to 10 NRS was most preferred as this is the scale most widely encountered in medical systems in the USA. For measuring pain interference, results suggested to adapt measures to have more items assessing pain interference in social activities.

## Figures and Tables

**Table 1:** Participant characteristics.

	Total Sample (*N* = 16) *n* (%) or Mean (*SD*)
Female	7 (44%)
Male	9 (56%)
Age (years)	54 (*SD* = 11) Range: 41 to 73
Chronic pain duration (years)	19.38 (*SD* = 10.68)
Worst pain intensity	4.56 (*SD* = 0.63)
Average pain intensity	3.69 (*SD* = 0.70)
Current pain intensity	3.13 (*SD* = 1.02)
Pain interference with day-to-day activities	4.0 (*SD* = 1.10)
Pain interference with work around the home	3.88 (*SD* = 1.20)
Pain interference with ability to participate in social activities	3.25 (*SD* = 1.61)
Pain interference with household chores	3.94 (*SD* = 1.12)

**Table 2:** Representative quotes on reactions to each explored psychological treatment for chronic pain.

Psychological Treatment	Representative Quotes
Behavioral	“I think that’s something that I could try.” “Yeah, that sounds like something I would like.” “If you can overdo things, you’re going to be hurting and stuff like that. Um, but to a degree depending on what your limits are. Yeah, it would be beneficial. I haven’t had suggestions. I’ll try yoga, you know things like that, or just try moving more.”
Cognitive	“I like cognitive behavioral therapy, uh, because, like you did say, our minds are very powerful, you know. Um, you know, and then we can find ourselves, maybe not consciously, but find ourselves being too hard on ourselves, you know, like I should be doing this or this should not be this way, or I should not be in so much pain. And that’s a lot of heavy burden that we’re having to carry when we could… we could approach it differently.” “It’s interesting. Yeah, I do that all the time. Positive stuff rather than negative. I don’t know if helps the pain or not, um, but… it’s hard to keep that negative out, you know.” “I use journaling for that, to try to get those feelings out of me. So, I’m not hanging onto something that’s poison in my body. You know the negativity that I’m going through.”
Mindfulness	“I’d like to learn more of the meditation techniques and stuff.” “Yeah, I think mindfulness is good. It’s in my opinion, it’s not easy, right. It takes a lot of practice. Um, but I do find that… at times it is helpful.” “I think it could benefit a lot of people, because there’s so much negativity around us all the time, you know, and it’s easy to get caught up in that.”
Therapeutic hypnosis	“It’s something that I would be interested in doing. Anything to learn another tool.” “I’m not sure how I feel about that. It’s interesting. I’m probably going to end up reading about it later because I’m not sure how I feel about it.” “I think that just the word ‘hypnosis’ is going to scare a lot of people, and because they don’t know about it… they just know the hypnosis is where they make you do things.”

**Table 3:** Representative quotes on the theme of stigmatization and invalidation.

Sub-Theme	Representative Quotes
Drug seeking	“They lump us all into a role of ‘we’re all bad people. We’re all just trying to get pain meds’ and that’s really not the case.” “Every time I go to the clinic for any kind of ailments, I’m always treated as if I’m there for pain meds.” “Any time you complain about pain, they’re just like, ‘Oh, here we go again! Somebody that’s out here looking for opiates.’”
Disregarded	“It’s really hard dealing with doctors when they don’t listen to you, especially when you have everything in charts and records that they can go back and look up.” “They x-ray you or MRI. They’re like, ‘Well, everything’s pretty normal. You shouldn’t have any pain.’ They just want to dismiss you and not believe you.” One participant who presented to the emergency room with severe pain, said doctors told them, “… that it was not anything.” The participant added, “They [the providers] couldn’t figure out what was going on until after they went through two different scans and found a clot and a kidney stone. And then they finally started listening to me; but dealing with them for two days before they actually added my breakthrough medicine where I could actually function… It was hard.”
Pain is not real	“One of the things I hate hearing the most, and I think it’s absolutely a horrible thing to do to a patient is go, ‘Oh well, I think you just have anxiety.’ I think that was so undermining and so degrading to a patient, because that’s using someone’s mental health against them and ignoring what they’re saying.”
Stigma of psychological treatments for chronic pain	“They want you to go through all kinds of uh mental … interventions. You know that really makes you feel like you are crazy, like they’re not believing you.” “… seems to me like it’s a push off. They don’t want to be with you anymore, because they’re not going to give you the drug you want… They’re not going to do this for you. So… they push you out the door to mental health.”

## Data Availability

Please reach out to the corroborating author regarding data availability.

## Abbreviations

The following abbreviations are used in this manuscript:

AI/AN	American Indian/Alaska Natives
YSU	Portland Area Indian Health Services—Yakama Service Unit
